# Utilization of traditional, complementary and alternative medicine and mental health among patients with chronic diseases in primary health care settings in Cambodia

**DOI:** 10.1186/s13033-017-0167-x

**Published:** 2017-09-22

**Authors:** Siyan Yi, Chanrith Ngin, Sovannary Tuot, Pheak Chhoun, Tyler Fleming, Carinne Brody

**Affiliations:** 1 KHANA Center for Population Health Research, No. 33, Street 71, Phnom Penh, Cambodia; 2 Public Health Program, Touro University California, Vallejo, CA USA

**Keywords:** Anxiety, Depression, Chronic diseases, Mental health, Traditional medicine, TCAM, Cambodia

## Abstract

**Background:**

Coping with chronic illnesses often involves major lifestyle changes that may lead to poor mental health. Furthermore, in order to treat the chronic conditions, many sufferers in Asia turn to traditional, complementary and alternative medicines (TCAM). This study explores prevalence of TCAM use and factors associated with anxiety and depressive symptoms among patients with chronic diseases in Cambodia.

**Methods:**

In 2015, this cross-sectional study was conducted with outpatients receiving treatment and care for chronic diseases in two urban and two rural primary health centers. Every eligible patient was randomly selected at the health centers using a systematic sampling procedure. Symptoms of anxiety and depression were assessed by using the Hospital Anxiety and Depression Scale (HADS). Multivariate logistic regression models were constructed to explore factors associated with anxiety and depressive symptoms.

**Results:**

The study participants included 1528 patients, of whom 77.2% were female, with a mean age of 46.5 years (SD = 15.3). After adjustment, patients with depressive symptoms remained significantly more likely to be in the age groups between 41 and 60 years old and to be married, separated/divorced or widowed compared to those without depressive symptoms. Regarding the use of TCAM, patients with depressive symptoms remained significantly more likely to report using an herbalist, practicing visualization and praying for own health, but less likely to report using vitamins or supplements in the past 12 months. For quality of life, patients with depressive symptoms remained significantly less likely to agree that they had enough energy for their everyday life and had enough money to meet their daily needs. Similar risk factors were also found to be significantly associated with anxiety symptoms.

**Conclusions:**

Cambodian patients with chronic diseases who experienced symptoms of anxiety or depression were more likely to report reduced quality of life, greater chronic disease-related stigma and more TCAM use. Given the potential interaction of TCAM, mental health and other chronic conditions, a history of TCAM use and mental health should be elicited in clinical practices in primary health care settings, particularly in developing countries.

## Background

Chronic diseases cause significant morbidity and mortality in Southeast Asia. In the last 10 years, deaths due to chronic diseases among those aged 10–54 increased by 10% [1]. Chronic diseases not only negatively impact health, but also cause undue burden due to unemployment, laborious treatments, restricted mobility and loss of independence [2–5]. Cambodia is not exempt from the effect of chronic diseases. From 2005 to 2015, the number of years lost due to chronic diseases increased by 10% for people aged 10–54 in the Kingdom [1]. A 2005 study found that 30% of participants in Siem Reap and 56% of participants in Kampong Cham suffered from some form of glucose intolerance, hypertension or both [6]. In a country torn apart by the Khmer Rouge regime, psychological disorders are also pervasive. In the 2012 Cambodian Mental Health Survey, 32% of women and 18% of men demonstrated symptoms of anxiety, and 20% of women and 10% of men demonstrated symptoms of depression [1, 7].

Past research from throughout Asia indicates that adults with chronic illnesses are more likely to experience anxiety and depression as comorbidities. Patients with ischemic heart disease had a higher anxiety score than those without heart disease in a 2014 study from Malaysia [8]. Moreover, anxiety and panic disorders were more prevalent in patients with chronic obstructive pulmonary disease (COPD) in Chiang Mai, Thailand, than in patients without any history of chronic respiratory disease [4].

Diminished quality of life and poorer health outcomes are commonly reported in chronic disease patients with mental health comorbidities. In Thailand, epileptic patients with a history of anxiety and depression reported a significantly decreased quality of life [3]. In a 2012 study, Lim et al. found that anxiety and depression among adults with chronic diseases in Singapore were associated with lower mental and physical functioning scores [9]. Notably, the researchers demonstrated that there is a multiplicative effect of chronic illnesses and anxiety and depression in decreasing quality of life. In a sample of Chinese adults with chronic obstructive pulmonary disorder, participants with anxiety and depression had a higher body mass index (BMI), degree of airflow obstruction, dyspnea and exercise capacity (BODE) index, shorter 6-min walk distance, more dyspnea and a greater quality of life score [10]. The evidence from Asian-wide studies reveals that chronic diseases interact with anxiety and depression to negatively impact mental and physical wellbeing [2–5, 9, 10]. However, there has been no study confirming this relationship in Cambodia.

People living with chronic diseases often feel stigmatized due to their conditions, in addition to experiencing anxiety and depression. More than 70% of people with epilepsy in China reported facing stigma in a 1995 study [11]. This is unsurprising, as studies have shown that over 60% of respondents in South Korea and over 85% in China disapprove of their children marrying someone with epilepsy [12, 13]. People living with hepatitis B in Malaysia reported fearing that disclosure of their status would result in being ostracized by the community [14]. Senior chronic disease sufferers in South Korea reportedly worried about losing their jobs as a result of their illnesses, and thought they would be judged as not adequately managing their own health [15]. In a study of people with diabetes in Singapore, 12% were classified as stigma positive if they believed that revelation of their diabetes diagnosis would result in differential treatment by their colleagues [16]. The fear of stigma causes many people to conceal their disease [17].

Like the effects of mental health, these high rates of perceived stigma negatively impacted patients’ reported quality of life [18]. Stigma positive individuals with diabetes in Singapore had a significantly higher number of median hospitalizations since 2001, as compared to stigma negative individuals [16]. They were also more likely to report their diabetes as affecting their work [16]. Over 25% of respondents in a study of hepatitis B sufferers in Malaysia felt unable to enjoy normal life activities since receiving their diagnosis [14]. Ng et al. [19] reported that high levels of perceived stigma about hepatitis B increased anxiety in those who had been newly diagnosed. Although there are many studies describing chronic disease related stigma and its effects on patients, no studies have further investigated how anxiety and depression may affect the stigma felt by those with chronic diseases.

Coping with chronic illnesses often involves major lifestyle changes. In order to treat pain associated with chronic diseases, many sufferers in Asia turn to traditional, complementary and alternative medicines (TCAM). TCAM use was reported at 64% among patients with chronic diseases in Malaysia [20], 60% among cancer patients in Thailand [21, 22] and 77% among adults with schizophrenia in Cambodia [23]. When chronic disease patients were asked about their medicine use in the previous year, 27% in Cambodia [24], 24% in Vietnam [24] and 23% in Singapore [25] reported using TCAM. A nationally representative household survey in Hong Kong found that people who consulted both western and traditional Chinese medicine practitioners were more likely to be older, be female, have a higher socio-economic status and have a chronic disease [26]. Furthermore, adults with arthritis, musculoskeletal disorders and stroke in Singapore had four times the odds of using TCAM as compared to patients without those conditions. Dissatisfaction with care and strong adherence to traditional beliefs also predicted TCAM use [25]. Use of TCAM among people with chronic diseases has been well established. However, how this relationship may vary based on anxiety and depression has not fully been explored.

The limited studies focusing on chronic diseases in Cambodia largely investigate disease management—how alternative medicines [24], early detection [6, 27] and access to health services [28] differ among specific chronic disease patients. We have found no studies that integrate both chronic disease and mental health experiences of Cambodians. This study, then, fills two important gaps in the literature: (1) it investigates the relationship between anxiety, depression and quality of life among Cambodians living with chronic illnesses and (2) it integrates the literatures of TCAM, chronic diseases and mental health to better understand how anxiety and depression alters the use of TCAM in this population. Understanding how quality of life and TCAM use varies for adults with anxiety and depression is essential to providing more comprehensive services to this population.

## Methods

### Study sites and participants

This cross-sectional survey was conducted as part of a multi-country study [24] with outpatients receiving care and treatment for chronic diseases in two urban and two rural primary health centers purposively selected based on the proportion of chronic disease patients who tend to frequent them from Phnom Penh, Kampong Cham and Siem Reap. The sample size included approximately 400 patients per primary health center, for an estimated prevalence of anxiety and depressive symptoms of 20% with a precision of ± 2%. Every eligible patient was randomly selected at the health centers using a systematic random sampling procedure.

A patient would be invited to participate in the study if he/she was: (1) aged older than 21 years; (2) able to communicate in Khmer; (3) willing to participate in the study and could provide a verbal informed consent; (4) able to present themselves on the day of the interview; (5) physically and mentally stable to participate in the study and (6) receiving care and treatment at the selected health centers for one or more of 20 chronic conditions including arthritis, asthma, cancer, cardiac arrhythmias, cardiac failure, COPD, coronary artery disease, diabetes mellitus, dyslipidaemia, epilepsy, gout and other musculoskeletal conditions, hypertension, kidney disease, liver disease, mental disorders, Parkinson disease, stomach and intestinal disease, stroke and thyroid disease [25, 26].

### Training and data collection

Data were collected in August and September 2014 by lay interviewers who underwent a 2-day training. The training covered necessary skills, including: understanding the study protocol, questionnaire and consent form; mastering interview techniques; becoming familiar with confidentiality and privacy protection and the questionnaire administration and pretesting. Quality control skills such as checking the questionnaires after administration and resolving issues that might arise during the fieldwork were also covered. Regular review sessions with interviewers were conducted during the survey period to review progress and communicate any problems or issues occurring during the data collection. The estimated time for each interview was approximately 30 min.

### Variables and measurements

#### Socio-demographic characteristics

Socio-demographic characteristics included age, gender, level of formal education completed, marital status, main occupation, main source of household income and type of neighborhood (urban or rural).

#### Use of TCAM

We used the I-CAM-Q, the international questionnaire to measure the use of TCAM that contains three sections. Section 1 asks about “visiting health care providers,” section 2 about the “use of herbal medicine and dietary supplements” and section 3 about “self-help practices.” The treatment modalities are presented in the form of a list, and respondents provide information on their usage over the previous 12 months (yes/no) [29].

#### Chronic disease related stigma

Chronic disease related stigma was measured using the Chronic Illness Anticipated Stigma Scale (CIASS) [30]. The CIASS measures anticipated stigma (i.e., expectations of prejudice, stereotyping and discrimination) among people living with chronic illnesses. It is a 12-item scale with three subscales differentiating among sources of anticipated stigma including friends, family members, colleagues and healthcare providers. Participants were asked to rate the likelihood that they would encounter these stigmatizing experiences in the future on a scale ranging from 1 (very unlikely) to 5 (very likely).

#### Quality of life

Quality of life was measured using the World Health Organization Quality-of-Life 8-question scale (WHOQOL-8) [31]. This consists of eight questions that were empirically derived from the WHOQOL-brief [32] addressing perceived aspects of the respondent’s quality of life in four principal domains: psychological, physical, social and environmental. Each question has five response options on a Likert-like scale, scored from 1 (worst) to 5 (best); the summed score has the potential range of 8–40, with higher scores indicating better quality of life.

#### Anxiety and depression

Symptoms of anxiety and depression were assessed using the Hospital Anxiety and Depression Scale (HADS). The HADS was developed to measure symptoms of anxiety and depression in somatically ill patients [33]. The scale is divided into an anxiety subscale (HADS-A) and a depression subscale (HADS-D), both containing seven intermingled items. The participants are asked to evaluate how they felt during the last week. Each item is scored between 0 and 3, where 0 is asymptomatic and 3 is considerable. The total score for each of the two subscales is between 0 (no sign of illness) and 21 (severe degree of anxiety or depression) [33, 34]. The HADS has been extensively used in the field of cancer [35] and several studies have shown good sensitivity and specificity with a cut-off score of 8+ for each of the two subscales [33, 36].

### Data analyses

EpiData version 3 was used for double data entry (Odense, Denmark) and SPSS version 22 (IBM Corporation, New York, USA) for all statistical analyses. In bivariate analyses, Student’s *t *test was used for continuous variables, and Chi square test (or Fisher’s exact test when a cell count was smaller than five) for categorical variables to compare socio-demographic characteristics; use of traditional healers, herbal medicines, dietary supplements and self-help practices; chronic illness anticipated stigma and quality of life among patients with and without anxiety and depressive symptoms.

A multivariate logistic regression model was separately constructed for anxiety and depressive symptoms. We first included all variables significantly associated with anxiety and depressive symptoms in bivariate analyses at a level of *P* value < 0.05 simultaneously in the model. Variables with a *P* value > 0.05 were then removed, and the model were refitted. The steps were repeated until all *P* values of the remaining variables were < 0.05 in the final model. Adjusted odds ratio (AOR) were obtained and presented with 95% confidence intervals (CI) and *P* values. Two-sided *P* values < 0.05 were used to indicate statistical significance.

### Ethical considerations

This study was approved by the National Ethics Committee for Health Research, the Ministry of Health in Cambodia (Ref: 0225NECHR). Participation in this study was voluntary, and a verbal consent was obtained from each participant after a detailed description of all study goals has been provided. Interviews were conducted in private locations, and confidentiality was protected by removing all personal identifiers from the questionnaires, field notes and the sampling frame. Participants were assigned a unique code. Researchers and data collectors were trained on confidentiality prior to data collection. Participants received a token of 3USD for time compensation.

## Results

### Socio-demographic characteristics

Data were collected from 1602 patients. Sixteen patients refused the participation due to their time constrains and were replaced by the next patient on the patient list. Patients with mental disorders (*n* = 74) were excluded from the analyses, leaving the final sample of 1528 patients. Of the included sample, 77.2% were female, and 62.2% were recruited from urban primary health centers with a mean age of 46.5 years (SD = 15.3). The majority of them (75.9%) were married, 64.4% had a formal education of 5 years or less and 96.5% were Buddhist. Almost half (48.0%) were a housewife or homemaker, and only 19.5% were currently employed. Their self-reported chronic conditions for which they were receiving the care and treatment included stomach and intestinal diseases (71.5%), arthritis (25.2%), hypertension (25.1%), heart disease (15.6%), COPD (12.2%), diabetes mellitus (11.8%), migraine (10.3%), gout and other musculoskeletal conditions (10.2%), kidney diseases (6.7%), asthma (2.7%), Parkinson’s disease (2.0%), dyslipidemia (2.0%), liver diseases (1.9%), cardiac arrhythmia (1.2%), thyroid (0.8%), stroke (0.4%), cancer (0.3%) and epilepsy (0.3%).

Comparisons of socio-demographic characteristics of the participants with and without anxiety and depressive symptoms are shown in Table 1. A significantly higher proportion of the participants with anxiety symptoms were older, female, separated or divorced, with lower level of education, currently unemployed or retired, receiving government or family support as main source of income and living in a rural area. Table 1 also shows that a significantly higher proportion of participants with depressive symptoms were older, widowed, with lower level of education, currently a housewife or homemaker or unemployed or retired, receiving formal salary as main source of income and living in a rural area.

**Table 1:** Comparison of socio-demographic characteristics of chronic disease patients with and without anxiety and depressive symptoms

Socio-demographic characteristics	Total anxiety score			Total depression score		
	< 8	≥ 8	*P* value*	< 8	≥ 8	*P* value*
Age			0.004			< 0.001
21–30	198 (35.4)	361 (64.6)		153 (27.4)	406 (72.6)	
31–40	4 (16.7)	20 (83.3)		4 (16.7)	20 (83.3)	
41–50	73 (26.6)	201 (73.4)		21 (7.7)	253 (92.3)	
51–60	97 (25.8)	279 (74.2)		20 (5.3)	356 (94.7)	
> 60	21 (27.5)	214 (72.5)		9 (3.1)	286 (96.9)	
Sex			< 0.001			0.69
Male	152 (43.6)	197 (56.4)		45 (12.9)	304 (87.1)	
Female	301 (25.5)	878 (81.7)		162 (13.7)	1017 (86.3)	
Marital status			0.001			< 0.001
Never married	51 (44.0)	65 (56.0)		50 (43.1)	66 (56.9)	
Married	342 (29.5)	817 (70.5)		144 (12.4)	1015 (87.6)	
Separated/divorced	14 (21.5)	51 (78.5)		8 (12.3)	58 (87.7)	
Widowed	46 (24.5)	142 (75.5)		5 (2.7)	183 (97.3)	
Formal education completed			< 0.001			< 0.001
No schooling	104 (22.1)	366 (77.9)		35 (7.4)	435 (92.6)	
1–5 years	147 (28.6)	367 (71.4)		55 (10.7)	459 (89.3)	
6–9 years	120 (35.8)	216 (64.3)		54 (16.1)	282 (83.9)	
10–12 years	53 (34.4)	101 (65.6)		27 (17.5)	127 (82.5)	
Higher education	27 (53.7)	26 (46.3)		36 (66.7)	18 (33.3)	
Current employment			< 0.001			< 0.001
Housewife/homemaker	217 (29.6)	577 (70.4)		72 (9.8)	662 (90.2)	
Unemployed/retired	13 (24.1)	41 (75.9)		6 (11.1)	48 (88.9)	
Employed	106 (35.6)	192 (64.4)		36 (12.1)	262 (87.9)	
Self-employed	69 (33.7)	136 (66.3)		51 (24.9)	154 (75.1)	
Students	23 (43.4)	30 (56.6)		32 (60.4)	21 (39.6)	
Other	25 (13.6)	159 (86.4)		10 (5.4)	174 (94.6)	
Main source of household income			< 0.001			< 0.001
Formal salary	47 (34.6)	89 (65.4)		9 (6.6)	127 (93.4)	
Family support	261 (31.1)	578 (68.9)		125 (14.9)	714 (85.1)	
NGO support	59 (43.4)	77 (56.6)		46 (33.8)	90 (66.2)	
Government support	18 (29.0)	44 (71.0)		14 (22.6)	48 (77.4)	
Other	68 (19.2)	287 (80.8)		13 (3.7)	342 (96.3)	
Type of neighborhood			< 0.001			< 0.001
Rural	237 (24.9)	713 (75.1)		34 (3.6)	916 (96.4)	
Urban	216 (37.4)	362 (62.6)		173 (29.9)	405 (70.1)	

Values are number of cases (%) for categorical variables and mean (± SD) for continuous variables

*SD* standard deviation

* Chi square test was used for categorical variables and t test for continuous variables

### Use of TCAM

As shown in Table 2, use of TCAM is common among patients with chronic conditions in this study. A significantly higher proportion of the participants with anxiety symptoms reported the use of an herbalist, practicing visualization (imagery) and praying for own health. A significantly lower proportion of participants with anxiety symptoms reported doing vigorous or moderate intensity sports or leisure activities in a typical week compared to those without anxiety symptoms. A significantly higher proportion of the participants with depressive symptoms reported the use of an herbalist, practicing of visualization (imagery) and praying for own health in the past 12 months compared to those without depressive symptoms. However, a significantly lower proportion of patients with depressive symptoms reported using homeopathic remedies and vitamins or supplements in the past 12 months and walking or cycling for at least 10 min per day and doing vigorous or moderate intensity sports or leisure activities in a typical week.

**Table 2:** Comparison of use of traditional, complementary and alternative medicine in past 12 months among chronic disease patients with and without anxiety and depressive symptoms

	Total anxiety score			Total depression score		
	< 8	≥ 8	*P* value*	< 8	≥ 8	*P* value*
Herbalist			0.001			< 0.001
No	397 (32.3)	832 (67.7)		199 (16.2)	1030 (83.8)	
Yes	56 (18.7)	243 (81.3)		8 (2.7)	291 (97.3)	
Spiritual healer			0.64			0.67
No	452 (29.7)	1071 (70.3)		206 (13.5)	1317 (86.5)	
Yes	1 (20.0)	4 (80.0)		1 (20.0)	4 (80.0)	
Acupuncturist			0.14			0.76
No	437 (30.0)	1018 (70.0)		198 (13.6)	1357 (86.4)	
Yes	16 (21.9)	57 (78.1)		9 (12.3)	64 (87.7)	
Chiropractor			0.91			0.15
No	436 (29.6)	1036 (70.4)		203 (13.8)	1269 (86.2)	
Yes	17 (30.4)	39 (64.6)		4 (7.1)	52 (92.9)	
Yoga practitioner			0.32			0.06
No	444 (29.5)	1061 (70.5)		207 (13.8)	1298 (86.2)	
Yes	9 (39.1)	14 (60.9)		0 (0.0)	23 (100.0)	
Massage therapist			0.62			0.67
No	441 (29.6)	1051 (70.4)		203 (13.6)	1289 (97.6)	
Yes	12 (33.3)	24 (66.7)		4 (11.1)	32 (88.9)	
Herbal medicine use			0.10			0.72
No	267 (31.3)	585 (68.7)		113 (13.3)	739 (86.7)	
Yes	186 (27.5)	490 (72.5)		94 (13.9)	582 (86.1)	
Using vitamins/supplements			0.68			< 0.001
No	347 (29.4)	834 (70.6)		103 (8.7)	1078 (91.3)	
Yes	106 (30.5)	241 (69.6)		104 (30.0)	243 (70.0)	
Using homeopathic remedies			0.86			< 0.001
No	437 (29.6)	1039 (70.4)		186 (12.6)	1290 (87.4)	
Yes	16 (30.8)	36 (69.2)		21 (40.4)	31 (59.6)	
Self-help practices—meditation			0.18			0.34
No	419 (30.1)	971 (69.9)		192 (13.8)	1198 (86.2)	
Yes	34 (24.6)	104 (75.4)		15 (10.9)	123 (89.1)	
Self-help practices—yoga			0.79			0.07
No	426 (29.7)	1007 (70.3)		200 (14.0)	1233 (86.0)	
Yes	27 (28.4)	68 (71.6)		7 (7.4)	88 (92.6)	
Self-help practices—qigong (energy healing)			0.21			0.39
No	446 (29.5)	1066 (70.5)		206 (13.6)	1306 (86.4)	
Yes	7 (43.8)	9 (56.2)		1 (6.3)	15 (93.7)	
Self-help practices—relaxation techniques			0.90			0.91
No	372 (29.7)	880 (70.3)		169 (13.5)	1083 (86.5)	
Yes	81 (29.3)	195 (70.7)		38 (13.8)	238 (86.2)	
Self-help practices—visualization (imagery)			< 0.001			< 0.001
No	378 (33.1)	764 (66.9)		190 (16.6)	952 (83.4)	
Yes	75 (19.4)	311 (80.6)		17 (4.4)	369 (95.6)	
Self-help practices—traditional healing ceremony			0.85			0.11
No	444 (29.7)	1052 (70.3)		206 (13.8)	1290 (86.2)	
Yes	9 (28.1)	23 (71.9)		1 (3.1)	31 (96.9)	
Self-help practices—praying for own health			< 0.001			< 0.001
No	346 (35.0)	642 (65.0)		184 (18.6)	804 (81.4)	
Yes	107 (19.8)	443 (80.2)		23 (4.3)	517 (95.7)	
Walk or use a bicycle for at least 10 min			0.28			< 0.001
No	286 (30.7)	647 (69.3)		75 (8.0)	858 (92.0)	
Yes	167 (28.1)	428 (71.9)		132 (22.2)	463 (77.8)	
Do vigorous intensity sports or leisure activities			0.01			< 0.001
No	415 (29.1)	1013 (70.9)		165 (11.6)	1363 (88.4)	
Yes	38 (41.8)	53 (58.2)		40 (44.0)	51 (56.0)	
Do moderate intensity sports or leisure activities			0.04			< 0.001
No	366 (28.6)	912 (71.4)		124 (9.7)	1154 (90.3)	
Yes	87 (34.8)	163 (65.2)		83 (33.2)	167 (66.8)	

Values are number of cases (%)

* Chi square test (or Fisher’s exact test when a cell count was smaller than five) was used

### Chronic illness anticipated stigma

Table 3 shows comparison of chronic illness anticipated stigma among patients with and without anxiety and depressive symptoms. A significantly higher proportion of patients with anxiety symptoms anticipated that a friend or family member will think that their illness is their fault; a friend or family member will not think as highly of them; a friend or family member will blame them; a friend or family member will be angry with them; someone at work will think that they cannot fulfill their work; someone at work will discriminate against them; a healthcare worker will blame them for not getting better and a healthcare worker will think that they are a bad patient. A significantly higher proportion of the participants with depressive symptoms reported that a friend or family member will blame them, their employer will assign a project to someone else and their employer will not promote them.

**Table 3:** Comparison of chronic illness anticipated stigma among patients with and without anxiety and depressive symptoms

	Total anxiety score			Total depression score		
	< 8	≥ 8	*P* value*	< 8	≥ 8	*P* value*
Friends/family members will think that your illness is your fault			< 0.001			0.09
No	432 (32.0)	916 (68.0)		190 (14.1)	1158 (85.9)	
Yes	21 (11.7)	159 (88.3)		17 (9.4)	163 (90.6)	
Friends/family members will not think as highly of you			< 0.001			0.13
No	433 (31.2)	956 (68.8)		194 (14.0)	1195 (86.0)	
Yes	20 (14.4)	919 (85.6)		13 (9.4)	126 (90.6)	
Friends/family members will blame you			< 0.001			0.02
No	434 (31.9)	926 (68.1)		194 (14.3)	1166 (85.7)	
Yes	19 (11.3)	149 (88.7)		13 (7.7)	155 (92.3)	
Friends/family members will be angry with you			< 0.001			0.02
No	437 (31.9)	934 (68.1)		195 (14.2)	1176 (85.8)	
Yes	16 (10.2)	141 (89.8)		12 (7.6)	145 (92.4)	
Someone at work will think that you cannot fulfill your work			0.03			0.19
No	449 (30.0)	1046 (70.0)		200 (13.4)	1295 (86.6)	
Yes	4 (12.1)	21 (87.9)		7 (21.2)	26 (78.8)	
Your employer will assign a project to someone else			0.14			0.03
No	448 (29.9)	1113 (70.8)		199 (13.3)	1300 (86.7)	
Yes	5 (17.2)	24 (82.8)		8 (27.6)	21 (72.4)	
Someone at work will discriminate against you			0.03			0.06
No	450 (30.0)	1051 (70.0)		200 (13.3)	1301 (86.7)	
Yes	3 (11.1)	24 (88.9)		7 (25.9)	24 (74.1)	
Your employer will not promote you			0.07			0.03
No	450 (29.9)	1053 (70.1)		200 (13.3)	1303 (86.7)	
Yes	3 (12.0)	22 (88.0)		7 (28.0)	18 (72.0)	
Healthcare workers will blame you for not getting better			0.03			0.89
No	440 (30.2)	1017 (69.8)		197 (13.5)	1260 (86.5)	
Yes	13 (18.3)	58 (81.7)		10 (14.1)	61 (85.9)	
Healthcare worker will be frustrated with you			0.18			0.57
No	432 (30.0)	1006 (70.0)		193 (13.4)	1245 (86.6)	
Yes	21 (23.3)	69 (76.7)		14 (18.6)	76 (84.4)	
Healthcare workers will give you poor care			0.11			0.70
No	441 (30.0)	1028 (70.0)		200 (13.6)	1269 (86.4)	
Yes	12 (20.3)	47 (79.7)		7 (11.9)	52 (88.1)	
Healthcare workers will think that you are a bad patient			0.03			0.70
No	443 (30.2)	1026 (69.8)		200 (13.6)	1269 (86.4)	
Yes	10 (16.9)	49 (83.1)		7 (11.9)	52 (88.1)	

Values are number of cases (%)

* Chi square test (or Fisher’s exact test when a cell count was smaller than five) was used

### Quality of life

Comparison of several items of quality of life among patients with and without anxiety and depressive symptoms are shown in Table 4. A significantly higher proportion of patients with anxiety symptoms reported that their quality of life was poor or very poor. They were significantly more likely to report that they were dissatisfied with their own health, themselves and the current living place conditions. They were also significantly less likely to report that they had enough energy for everyday life and money to meet daily needs. Similarly, a significantly higher proportion of patients with depressive symptoms reported that their quality of life was poor or very poor; that they were dissatisfied with their own health, ability to perform daily activities, themselves, personal relationships and current living place conditions; and that they did not have enough energy for everyday life and money to meet daily needs.

**Table 4:** Comparison of quality of life among chronic disease patients with and without anxiety and depressive symptoms

Quality of life	Total anxiety score			Total depression score		
	< 8	≥ 8	*P* value*	< 8	≥ 8	*P* value*
Self-rated quality of life			< 0.001			< 0.001
Poor/very poor	37 (19.4)	154 (80.6)		8 (4.2)	183 (95.8)	
Neither poor nor good	175 (27.5)	461 (72.5)		87 (13.7)	449 (86.3)	
Good/very good	241 (34.4)	460 (65.6)		112 (16.0)	589 (84.0)	
Satisfaction with your own health			< 0.001			0.001
Dissatisfied	1 (1.8)	54 (98.2)		3 (5.5)	52 (94.5)	
Neutral	205 (28.5)	515 (71.5)		77 (10.7)	643 (89.3)	
Satisfied	247 (32.8)	506 (67.2)		127 (16.9)	626 (83.1)	
Having enough energy for your everyday life			0.001			< 0.001
Not at all/a little	135 (25.1)	402 (74.9)		9 (1.7)	528 (98.3)	
Moderately	153 (28.9)	376 (71.1)		54 (10.2)	475 (89.8)	
Mostly/completely	165 (36.7)	297 (64.3)		144 (31.2)	318 (68.8)	
Satisfaction with ability to perform your daily activities			0.38			< 0.001
Dissatisfied	13 (22.4)	45 (77.6)		12 (20.7)	46 (79.3)	
Neutral	181 (29.0)	443 (71.0)		24 (3.8)	600 (96.2)	
Satisfied	269 (30.6)	587 (69.4)		171 (20.2)	675 (79.8)	
Satisfaction with yourself			0.001			<0.001
Dissatisfied	2 (4.7)	41 (95.3)		2 (4.7)	41 (95.3)	
Neutral	222 (30.3)	511 (69.7)		77 (10.5)	656 (89.5)	
Satisfied	229 (30.5)	528 (69.5)		128 (17.0)	624 (83.0)	
Satisfaction with your personal relationships			0.17			< 0.001
Dissatisfied	5 (25.0)	15 (75.0)		5 (25.0)	15 (75.0)	
Neutral	204 (32.2)	429 (67.8)		59 (9.3)	574 (90.7)	
Satisfied	244 (27.9)	631 (72.1)		143 (16.3)	732 (83.7)	
Having enough money to meet your daily needs			< 0.001			< 0.001
Not at all/a little	271 (24.6)	831 (75.4)		88 (8.0)	1014 (92.0)	
Moderately	177 (42.3)	241 (57.7)		113 (27.0)	305 (73.0)	
Mostly/completely	5 (62.5)	3 (37.5)		6 (75.0)	2 (25.0)	
Satisfaction with your currently living place conditions			0.03			< 0.001
Dissatisfied	25 (17.2)	105 (80.8)		22 (16.9)	108 (83.1)	
Neutral	120 (30.5)	274 (69.5)		27 (6.9)	367 (93.1)	
Satisfied	308 (30.7)	696 (69.3)		158 (15.7)	846 (84.3)	

Values are number of cases (%)

* Chi square test (or Fisher’s exact test when a cell count was smaller than five) was used

### Factors associated with anxiety and depressive symptoms

The results of multivariate logistic regression analyses are shown in Table 5. After controlling for other covariates, patients with anxiety symptoms remained significantly more likely to be female (AOR = 2.17, 95% CI 1.61–2.93) and less likely to report having completed 6–9 years of formal education (AOR = 0.65, 95% CI 0.46–0.91). Regarding the use of TCAM, patients with anxiety symptoms remained significantly more likely to report using an herbalist (AOR = 1.65, 95% CI 1.17–2.31), practicing visualization (AOR = 2.35, 95% CI 1.62–3.41) and praying for own health (AOR = 1.81, 95% CI 1.33–2.48) in the past 12 months. Moreover, they were significantly less likely to agree that they were satisfied with their own health (AOR = 0.11, 95% CI 0.01–0.81), satisfied with themselves (AOR = 0.19, 95% CI 0.04–0.89), had enough energy for their everyday life (AOR = 0.57, 95% CI 0.39–0.82), had enough money to meet their daily needs (AOR = 0.55, 95% CI 0.41–0.73) and were satisfied with the conditions of their current living place conditions (AOR = 0.58, 95% CI 0.35–0.96).

**Table 5:** Factors associated with anxiety and depressive symptoms in multivariate logistic regression models

Variables in final models^a^	Anxiety score (< 8 vs. ≥ 8)		Depression score (< 8 vs. ≥ 8)	
	AOR (95% CI)	*P* value	AOR (95% CI)	*P* value
Age				
21–30			Reference	
31–40			0.71 (0.18–2.78)	0.62
41–50			2.43 (1.34–4.43)	0.004
51–60			2.37 (1.30–4.32)	0.005
> 60			2.26 (1.00–5.13)	0.05
Gender				
Male	Reference			
Female	2.17 (1.61–2.93)	<0.001		
Marital status				
Never married			Reference	
Married			4.96 (2.65–9.28)	< 0.001
Separated/divorced			3.05 (1.01–9.24)	0.04
Widowed			11.84 (3.54–39.63)	< 0.001
Formal education completed				
No schooling	Reference			
1–5 years	0.79 (0.58–1.07)	0.13		
6–9 years	0.65 (0.46–0.91)	0.01		
10–12 years	0.75 (0.49–1.17)	0.21		
Higher education	0.53 (0.28–1.01)	0.05		
Using an herbalist				
No	Reference		Reference	
Yes	1.65 (1.17–2.31)	0.004	3.68 (1.61–8.43)	0.002
Self-help practices—visualization (imagery)				
No	Reference		Reference	
Yes	2.35 (1.62–3.41)	<0.001	5.02 (2.34–10.75)	<0.001
Self-help practices—praying for own health				
No	Reference		Reference	
Yes	1.81 (1.33–2.48)	<0.001	4.25 (2.11–8.57)	<0.001
Using vitamins/supplements				
No			Reference	
Yes			0.50 (0.33–0.85)	0.001
Satisfaction with your own health				
Dissatisfied	Reference			
Neutral	0.14 (0.02–1.09)	0.06		
Satisfied	0.11 (0.01–0.81)	0.03		
Satisfaction with yourself				
Dissatisfied	Reference			
Neutral	0.19 (0.04–0.89)	0.04		
Satisfied	0.23 (0.05–1.06)	0.06		
Having enough energy for your everyday life				
Not at all/a little	Reference		Reference	
Moderately	0.92 (0.68–1.24)	0.58	0.18 (0.08–0.35)	<0.001
Mostly/completely	0.57 (0.39–0.82)	0.002	0.04 (0.02–0.08)	<0.001
Having enough money to meet your needs				
Not at all/a little	Reference		Reference	
Moderately	0.55 (0.41–0.73)	<0.001	0.45 (0.30–0.69)	0.002
Mostly/completely	0.61 (0.14–2.74)	0.52	0.32 (0.05–1.99)	0.22
Satisfaction with your current living place conditions				
Dissatisfied	Reference			
Neutral	0.50 (0.29–0.85)	0.01		
Satisfied	0.58 (0.35–0.96)	0.04		

*AOR* adjusted odds ratio, *CI* confidence interval

^a^Variables in the table were the ones that remained statistically associated with anxiety and depressive symptoms in the final multivariate logistic regression model after several steps of model fitting

Table 5 also shows that patients with depressive symptoms remained significantly more likely to be in the age group of 41–50 (AOR = 2.43, 95% CI 1.34–4.43) and 51–60 (AOR = 2.37, 95% CI 1.30–4.32) and to be married (AOR = 4.96, 95% CI 2.65–9.28), separated/divorced (AOR = 3.05, 95% CI 1.01–9.24) or widowed (AOR = 11.84, 95% CI 3.54–39.63) compared to those without depressive symptoms. Patients with depressive symptoms remained significantly more likely to report using an herbalist (AOR = 3.68, 95% CI 1.61–8.43), practicing visualization (AOR = 5.02, 95% CI 2.34–10.75) and praying for their own health (AOR = 4.25, 95% CI 2.11–8.57), but less likely to report using vitamins or supplements (AOR = 0.50, 95% CI 0.33–0.85) in the past 12 months. Regarding quality of life, patients with depressive symptoms remained significantly less likely to agree that they had enough energy for their everyday life (AOR = 0.18, 95% CI 0.08–0.35) and had enough money to meet their daily needs (AOR = 0.45, 95% CI 0.30–0.69).

## Discussion

The results of this study are consistent with other studies in the region [2, 3, 9, 10, 37], which have found that patients with anxiety and depressive symptoms are likely to utilize TCAM, report a low quality of life and great stigma related to their chronic conditions. Like preceding literature, our study found that being female did statically increase the chances of having anxiety symptoms, but our study found no association between depressive symptoms and gender, unlike other work in the field. This work also reinforced the theme that anxiety and depression can worsen chronic disease outcomes. Our study showed that those with anxiety and depressive symptoms were less likely to report having less energy, and that they lacked the financial means of support.

Like a 2012 study of COPD sufferers [10] and a 2014 study of end-stage renal disease patients [2] in China, we found that being female was significantly associated with anxiety symptoms. Whereas Hou et al. [2] found that being male was associated with depression, our study differs in that we did not find that any gender was statistically significantly associated with depressive symptoms. Women in Cambodian society are responsible for the general family welfare. That women with chronic diseases are more likely to suffer anxiety symptoms may be related to the stress around simultaneously balancing a chronic disease and family caretaking. While women worldwide typically report higher rates of depression, in our study this was not the case. In Cambodia, as a result of the genocide, rates of depression and trauma-related mental health disorders is higher in affected populations across both genders—higher even compared to other post-conflict countries [38]. In our study, the mean age of those reporting higher rates of depressive symptoms was significantly higher than those reporting lower rates which may indicate that participants in this group are more likely to have been affected by the 1975–1979 genocide. This may account for the lack of the gender difference in contrast to most other places in the world.

Other studies demonstrated that chronic disease patients with depressive symptoms were more likely to have lower incomes [8, 10, 39]. Although our study did not include level of income as a covariate, one of the quality of life measures was whether or not the respondent had enough money to meet daily needs. Participants with anxiety and depressive symptoms were both significantly less likely to report having moderately enough money to meet their daily needs. Although we cannot infer that chronic diseases caused anxiety or depressive symptoms among study participants, the effect on income may reflect that symptoms of anxiety or depression compound the work-related challenges associated with chronic diseases. It may be even more difficult for chronic disease sufferers with anxiety or depression to maintain stable employment. This association may also represent the stress associated with finding the resources to pay for the maintenance of a chronic disease. Out-of-pocket payments for health care are the largest expense for households in Cambodia after food expenditures; this places an enormous burden on poorer families [40].

One of the most important findings from the literature was that anxiety and depression not only negatively impact mental health of patients with chronic diseases, but also worsened health outcomes [9, 10]. Our study may have reinforced this conclusion; respondents with anxiety and depressive symptoms were significantly less likely to report having enough energy for their everyday life. In bivariate analyses, participants with depressive symptoms were also significantly less likely to get involved in physical activities such as walking or using a bicycle and doing vigorous or moderate intensity sports or leisure activities. While our study design makes it difficult to know for certain if the chronic disease preceded the anxiety and depressive symptoms or the other way around, experiencing comorbid depressive symptoms was the cause of less energy and physical activity, as the greater Asian literature predicted [9, 10].

In the 2016 study about TCAM use in Cambodia, Vietnam and Thailand, Peltzer et al. [24] reported that over 20% of Cambodian participants visited an herbalist in the last year. Almost half reported using an herbal medicine, and over 22% took vitamins or supplements in the last year. Over 35% of Cambodians prayed for their own health; almost one-third used visualization techniques and almost one-fifth used relaxation techniques to alleviate symptoms [24]. In multivariate analyses of all participants from the three countries, having two or more chronic conditions was associated with use of TCAM products [24]. Lee et al. [25] found that TCAM use was significantly associated with arthritis, musculoskeletal disorders and stroke in a Singaporean study population. Our study adds further understanding to factors associated with TCAM use and patients with chronic diseases. We found that respondents who reported anxiety or depressive symptoms were more likely to use an herbalist, practice visualization and pray for their own health in the last year. Participants who had depressive symptoms were also more likely to report using vitamins or supplements in the last year.

Other studies found that TCAM use was related to dissatisfaction of medical care [25]. Feeling depressed or anxious about one’s life may compound frustrations with one’s chronic disease. If respondents felt more anxious or depressed about their condition, then it could lead them to seek alternative methods of disease treatment and pain alleviation. Our study confirmed that patients with anxiety and depressive symptoms reported worse quality of life and less energy to conduct their everyday lives, both of which could impact a patient’s attitude about the assigned medical treatment.

## Limitations of the study

There are several important limitations to this study. This was a cross-sectional study and therefore, we are unable to draw causal inferences from our findings. Second, all measures were self-reported and not diagnosed by a medical or mental health professional. As a result, there may be biases in reporting that may lead to both underreporting and over-reporting of certain variables and also misclassification of disease and non-disease. This misclassification is likely to be unevenly distributed between diseases that are easier to self-diagnose such as gastrointestinal disorders and those that are more difficult such as cancers. We used validated scales to measure both outcomes to address this problem. All scales used in this study were developed by international panels that included Cambodian and/or Southeast Asian participants and have been successfully used in Cambodian populations. But despite successful usage of these scales, there may be linguistic and cultural factors that can affect the accuracy of such components as cut-points for anxiety and depressive symptoms. The final limitation concerns the generalizability of the findings to patients in other settings because our study participants were recruited from only two rural and two urban primary health centers.

## Conclusions

This study helps to understand more about how anxiety and depression relate to motivation to seek and utilize TCAM among patients with chronic diseases and the ways in which they use TCAM. Patients with anxiety symptoms reported more stigmatization about their disease from various sources including family, friends, work and health providers. Patients with anxiety and depressive symptoms were found to utilize herbalists, visualization and prayer, while those with depressive symptoms also had an increased utilization of vitamins and supplements.

The findings are important because healthcare providers need to ask key questions during the history and physical examination when treating patients to understand what types of treatments the patients have previously tried or are utilizing. This information is important so that the healthcare system can address the full scope of the patient’s complaints while working with them to craft a plan that will take the patient’s desires for treatment, treatment methods and means into account especially if the patient has anxiety or depression. Furthermore, results of this study suggest that, in routine visits for chronic medical conditions (such as diabetes for example), a quick screen for anxiety or depressive symptoms and utilization of TCAM would be warranted.

Future areas of study would include the association between income and depression and anxiety linked to chronic diseases. This study found that those with anxiety and depressive symptoms reported more perceived financial instability and solvency. In Cambodia, most medical expenses are paid for out of pocket and are the second highest household expense. A study examining the role of cost of care and poverty level leading to TCAM utilization might further shed light on the field. Additionally, the study sample, though random in design, was overwhelmingly female; thus a study examining the use of TCAM by gender and for what reason might also contribute to this body of knowledge.

## Abbreviations

AOR: adjusted odds ratio
CI: confidence interval
CIASS: Chronic Illness Anticipated Stigma Scale
HADS: Hospital Anxiety and Depression Scale
HIV: human immunodeficiency virus
COPD: chronic obstructive pulmonary disease
NECHR: National Ethics Committee for Health Research
NGO: non-governmental organization
SD: standard deviation
WHO: World Health Organization
SD: standard deviation
TCAM: traditional, complementary and alternative medicine
WHOQOL: World Health Organization Quality-of-Life scale
